# Laboratory Rat Vaginal Cytology: A Quick Method of Staging Unstained Cells

**DOI:** 10.7759/cureus.86966

**Published:** 2025-06-29

**Authors:** Shreya V. K., Ramendra K Raman, Shishir Kumar, Bindhu S, Juveriya Farooq, Nadira Noushida

**Affiliations:** 1 Department of Anatomy, Yenepoya Medical College, Yenepoya Deemed to be University, Mangalore, IND; 2 Clinical Anatomy, Mayo Institute of Medical Sciences, Lucknow, IND; 3 Anatomy, Institute of Medical Sciences & SUM Hospital, Siksha 'O' Anusandhan Deemed to be University, Bhubaneswar, IND; 4 Department of Anatomy, K. S. Hegde Medical Academy, Nitte Deemed to be University, Mangalore, IND; 5 Pharmacology, Yenepoya Pharmacy College and Research Centre, Yenepoya Deemed to be University, Mangalore, IND; 6 Department of Pharmacology, Yenepoya Medical College, Yenepoya Deemed to be University, Mangalore, IND

**Keywords:** dioestrus, estrous, metestrus, proestrus, rat estrous phase, vaginal cytology

## Abstract

Background

The estrous cycle in female laboratory rats is a critical parameter in reproductive and toxicological research, traditionally assessed through stained vaginal cytology. However, staining procedures can be time-consuming, costly, and less feasible in resource-limited settings. This study evaluates the efficacy of unstained vaginal cytology as a rapid, economical alternative for staging the estrous cycle in female albino Wistar rats.

Methods

Vaginal smears were collected daily from 30-day-old female albino Wistar rats for 15 consecutive days using saline lavage. Unstained smears were examined under light microscopy to identify the predominant cell types - cornified epithelial cells, nucleated epithelial cells, and leukocytes - and to classify the estrous phases (proestrus, estrus, metestrus, diestrus).

Results

Distinct cytological profiles corresponding to each estrous phase were reliably observed without staining. Proestrus featured predominantly nucleated epithelial cells, estrus was marked by abundant cornified epithelial cells, metestrus showed increased leukocytes, and diestrus was characterized by a predominance of leukocytes with fewer epithelial cells. The average cycle duration was four to five days. The unstained method provided clear, rapid identification of cycle phases comparable to traditional stained techniques.

Conclusion

Unstained vaginal cytology offers a simple, cost-effective, and reliable method for estrous cycle staging in female albino Wistar rats. This approach is particularly suitable for laboratories with limited resources, facilitating reproductive and toxicological research without compromising accuracy.

## Introduction

The estrous cycle in female laboratory rats is a recurring reproductive cycle lasting approximately four to five days, encompassing four distinct stages: proestrus, estrus, metestrus, and diestrus. These stages are characterized by specific morphological changes in the vaginal epithelium, which can be assessed via vaginal cytology to determine the phase of the cycle accurately [1,2]. Vaginal cytology provides an important, minimally invasive method for staging the estrous cycle and is widely used in biomedical research, toxicology, and reproductive studies to monitor hormonal fluctuations and fertility status [3,4].

While traditional cytological assessment often involves staining techniques such as the Shorr method, these procedures can be time-consuming and costly and require additional resources that may not be readily available in all laboratory settings, particularly in resource-limited environments [5]. By contrast, unstained vaginal smears observed under light microscopy allow the rapid identification of key cellular types - cornified epithelial cells, nucleated epithelial cells, and leukocytes - enabling quick staging of the estrous phases without the need for staining [1,2].

Despite the potential of unstained smear evaluation, inconsistencies and ambiguities remain in distinguishing specific cell types and estrous phases, especially for untrained personnel or when resource constraints limit the use of advanced staining protocols. Thus, there is a need to establish a simple, economical, and reliable method for estrous cycle staging based on unstained vaginal cytology that can be applied efficiently in diverse research settings [4,5].

This study aims to evaluate the cellular characteristics in unstained vaginal smears from female albino Wistar rats and provide a quick, cost-effective protocol for identifying the estrous cycle stages, facilitating research where conventional staining techniques are impractical.

## Materials and methods

Study design

This prospective descriptive study was conducted to evaluate unstained vaginal cytology as a rapid method for staging the estrous cycle in female albino Wistar rats. The study was conducted over a period of 15 consecutive days. The research was carried out at the institutional animal facility of Yenepoya Medical College in Mangalore, India, which is equipped with controlled conditions conducive to maintaining the health and well-being of the animals. The rats were housed at a constant temperature of 22 ± 2°C, with 50-60% relative humidity, and were kept on a 12-hour light/dark cycle. This study was performed under ethical approval (approval no.: YU/IAEC/09/2023), ensuring compliance with national guidelines for laboratory animal care.

However, due to resource constraints and ethical considerations, a sample size of 15 rats was chosen. This number was deemed sufficient to obtain reliable qualitative data while balancing the feasibility of the study and minimizing animal usage. The selected sample size aligns with the common practices for observational studies of this nature, where a smaller sample can still provide valuable insights, particularly when used for descriptive and preliminary data gathering.

Sample size calculation

The initial sample size for this study was calculated using a standard formula for estimating proportions with the following parameters: confidence level: 95% (Z = 1.96); estimated proportion: 0.5 (for maximum variability); and margin of error: 0.05 (5%)

Based on this calculation, the required sample size was approximately 385 rats. However, due to resource constraints and ethical considerations, a sample size of 15 rats was selected. This number was deemed sufficient to obtain reliable qualitative data while balancing feasibility and minimizing animal usage.

Animals and husbandry

Thirty-day-old female albino Wistar rats were obtained from the institutional animal facility and acclimatized for one week prior to the study. Animals were housed under controlled conditions with a temperature of 22 ± 2°C, relative humidity of 50-60%, and a 12-hour light/dark cycle. Rats had ad libitum access to a standard pellet diet and filtered water.

Ethical approval

The study protocol was approved by the Institutional Animal Ethics Committee of Yenepoya Medical College (approval no.: YU/IAEC/09/2023). All procedures complied with national guidelines for laboratory animal care.

Vaginal sample collection

Vaginal cytology samples were collected daily for 15 consecutive days at the same time each morning (9:00-10:00 AM) to reduce hormonal cycle variability. A sterile plastic pipette was used to gently instill approximately 0.1 mL of sterile physiological saline (0.9% NaCl) into the vaginal canal, inserting the pipette tip 1-2 mm to avoid cervical irritation. The saline was flushed and aspirated repeatedly until the lavage fluid became turbid, indicating sufficient cell recovery.

Each rat was sampled with a new sterile pipette tip, discarded immediately after use to prevent cross-contamination. Pipette tips contacting external areas such as the anus were discarded and replaced.

Slide preparation and cytological analysis

A drop of the turbid lavage fluid was placed on a clean, labeled glass slide and immediately covered with a cover slip. Slides were examined within one hour under a light microscope using 10× and 40× objectives.

The relative proportions of cornified epithelial cells, nucleated epithelial cells, and leukocytes were qualitatively assessed to classify the estrous cycle phase based on established cytological criteria.

The observations in this study were made by the primary researcher, who was trained in vaginal cytology and microscopy techniques. The observations were not conducted by a blinded expert, which could potentially introduce observer bias. To mitigate this, the researcher adhered to established cytological criteria for identifying the estrous phases and followed a consistent procedure throughout the study.

Furthermore, the findings and conclusions of the cytological observations were discussed with a team of experienced researchers and experts in the field of reproductive toxicology to ensure that the interpretations were accurate and aligned with existing literature. This collaborative approach aimed to validate the observations and minimize any subjective bias in the interpretation of the data.

Statistical analysis

This study was descriptive and observational. Cellular compositions were recorded and qualitatively assessed for each estrous phase. Data are presented as mean proportions with standard deviations where applicable. No formal statistical comparisons were performed due to the qualitative nature of the study.

## Results

**Figure 1:**
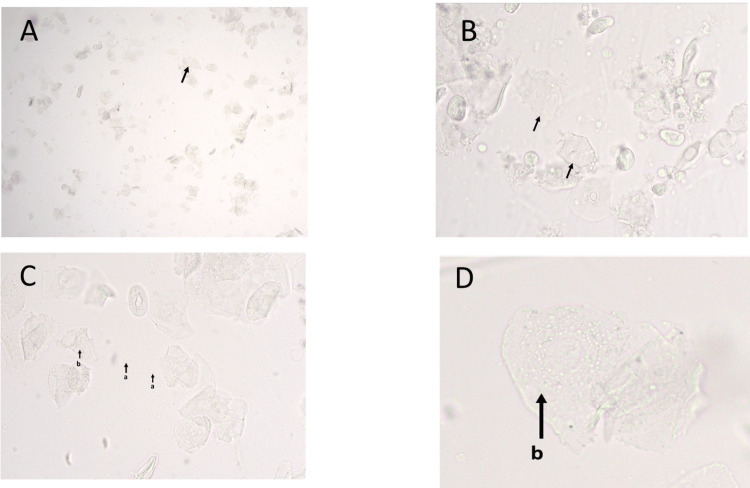
Microscopic images of unstained vaginal cytology smears from female albino Wistar rats showing characteristic cellular features of the estrous phase. Panel A: Low magnification showing predominantly non-nucleated epithelial cells in estrous phase (10X objective). Panel B: Higher magnification of estrous showing clusters of non-nucleated epithelial cells (40X objective). Panel C: Estrous phase showing abundant cornified epithelial cells(b) and free bacteria in background(a) (40X objective). Panel D: High magnification of estrus showing angular epithelial cells with faint “ghost” nuclei and cytoplasmic granules(b) (40X objective).

**Figure 2:**
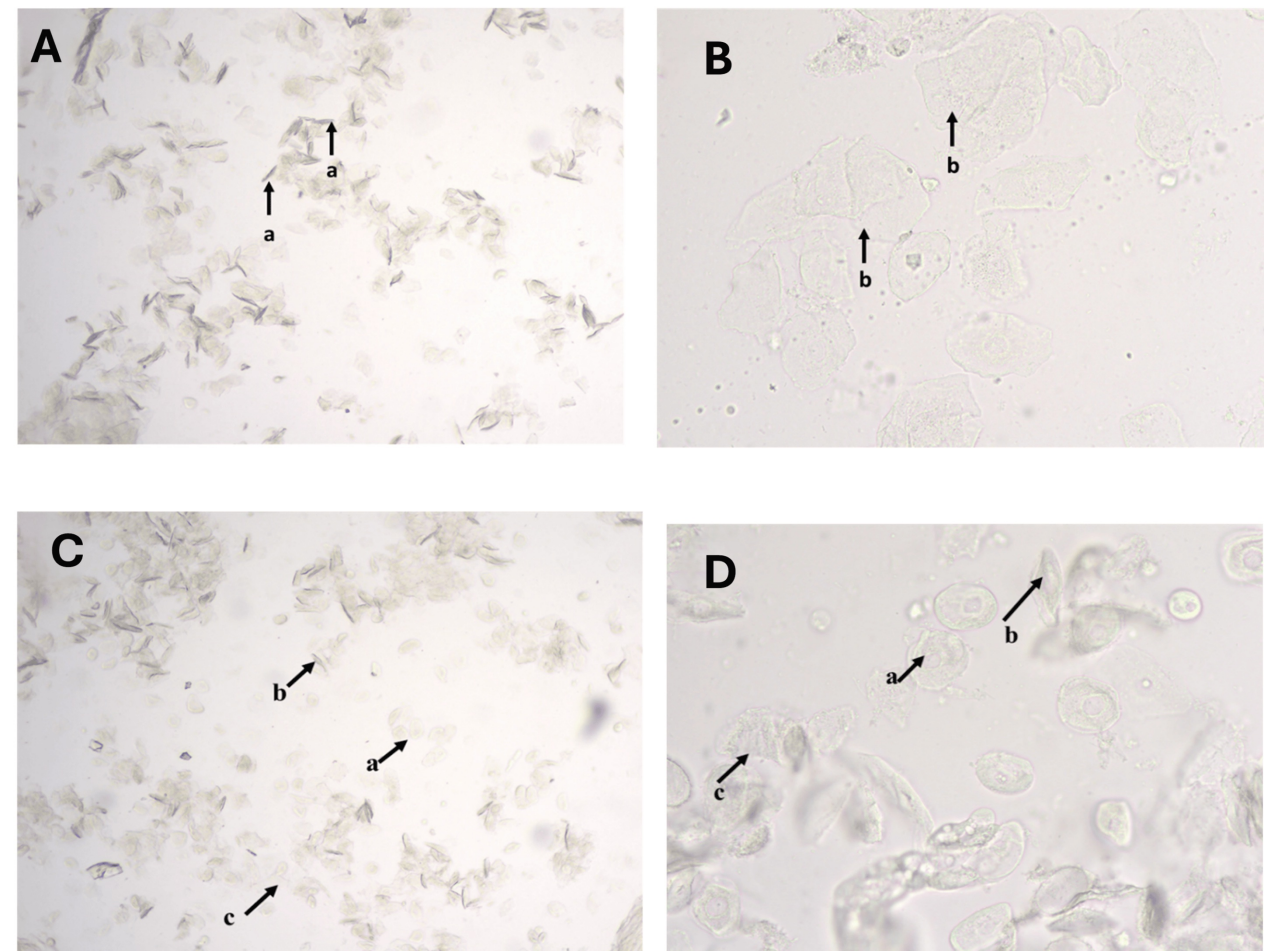
Unstained vaginal cytology smears under light microscopy illustrating cellular features during the estrus phase in female albino Wistar rats. Panel A: Low magnification showing keratin bars(a) (10X objective). Panel B: Higher magnification of estrus cells showing non-nucleated epithelial cells(b) (40X objective). Panel C: Late estrous phase showing nucleated epithelial cells(a), Spindle shaped nucleated epithelial cells(b) and non-nucleated epithelial cells(c) (10X objective). Panel D: High magnification of late estrous phase showing nucleated epithelial cells(a), Spindle shaped nucleated epithelial cells(b) and non-nucleated epithelial cells(c) (40X objective).

**Figure 3:**
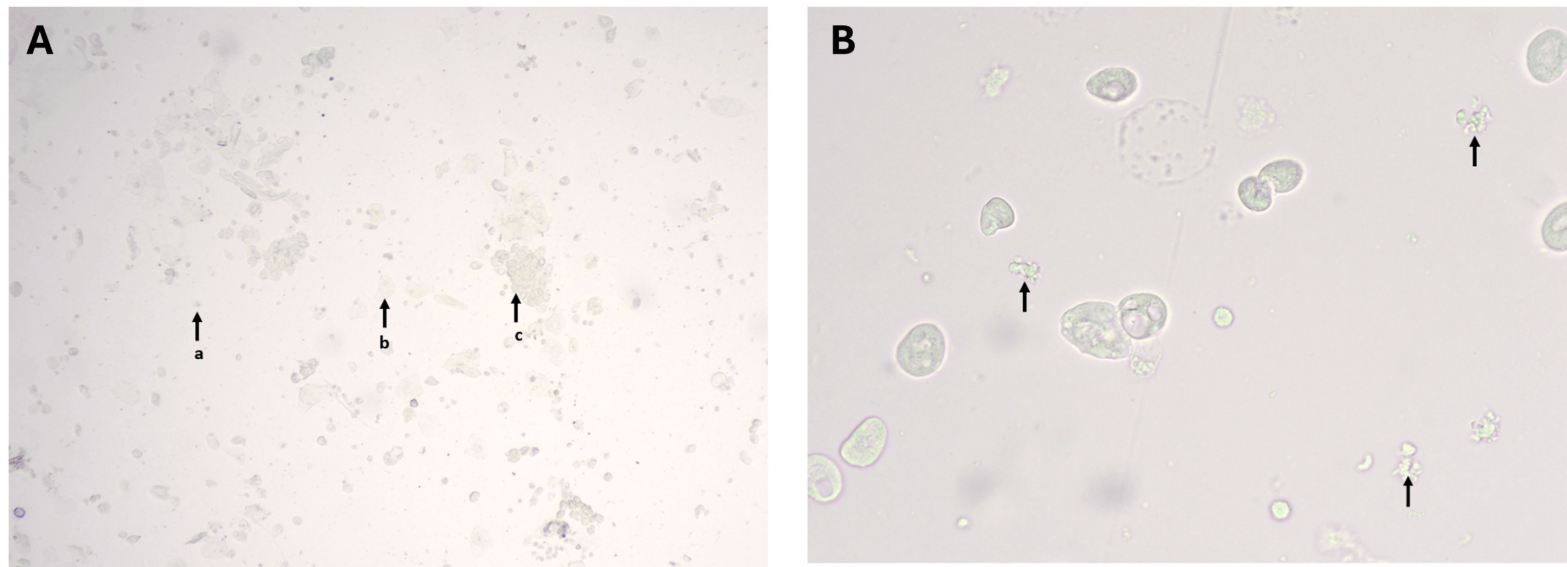
Unstained vaginal cytology smears depicting the metaestrous phase in female albino Wistar rats. Panel A: Light microscopy(10X) image showing: leucocyte(a), epithelial cells(b) and leucocytes seen in clumps(c) Panel B: Higher magnification(40X) showing leukocytes and ruptured neutrophils (black arrow)

**Figure 4:**
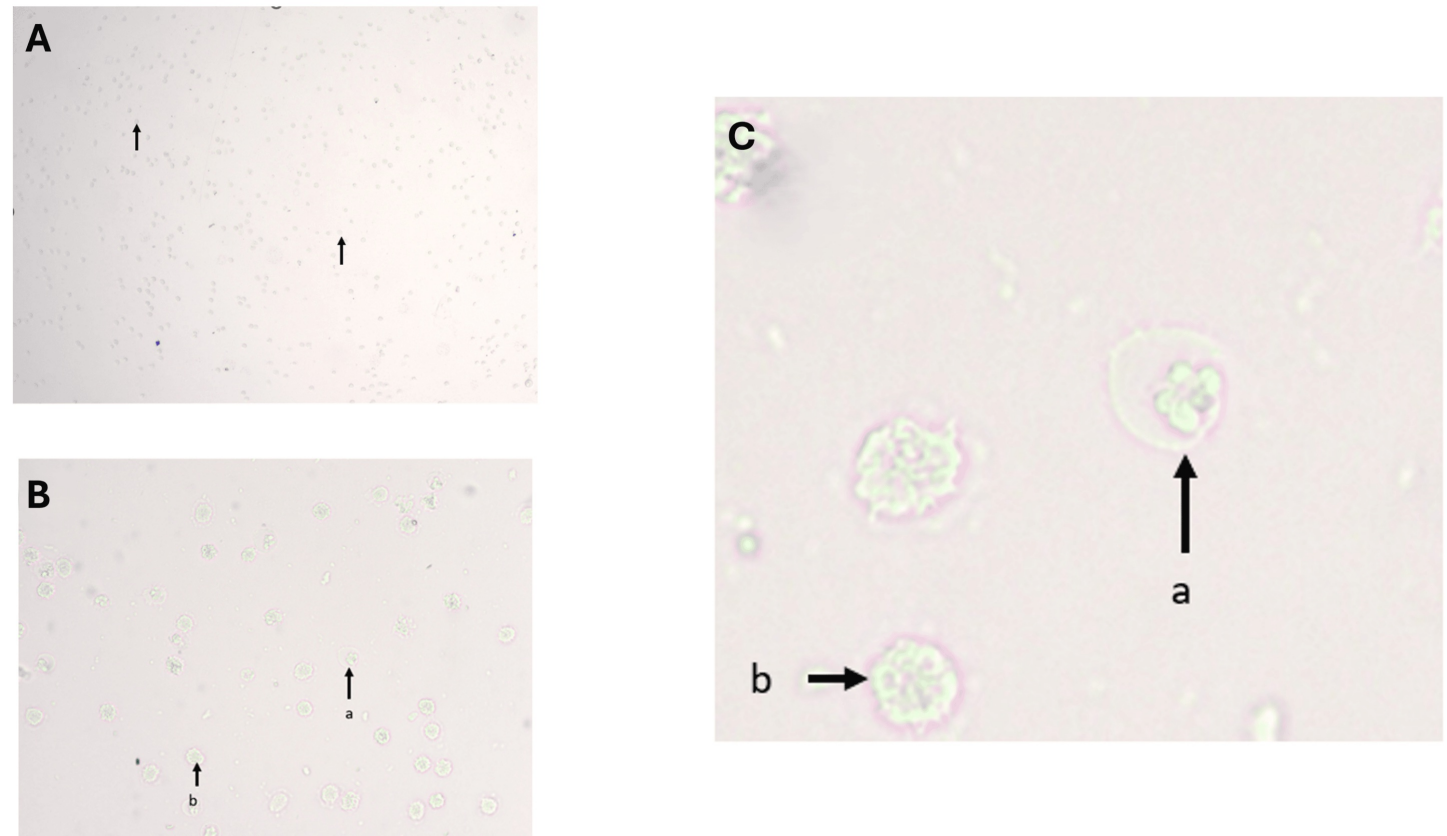
Unstained vaginal cytology smears depicting leukocyte predominance in the diestrus phase of female albino Wistar rats. Panel A: Light microscopy image showing scattered leukocytes indicated by arrows. Panel B: Higher magnification revealing individual leukocytes with clear morphology; arrows mark representative cells. Panel C: Close-up view of leukocytes: arrow "a" points to a neutrophil with a multilobed nucleus, arrow "b" shows a lymphocyte.

The estrous cycle in female albino Wistar rats was clearly classified into four distinct phases - proestrus, estrus, metestrus, and diestrus - based on unstained vaginal cytology smears (Figures 1A-1D, 2A-2D, 3A-3B, 4A-4C). During the proestrus phase, vaginal smears predominantly contained small, rounded nucleated epithelial cells, appearing in clusters or sheets (Figures 1A, 1B, 4B). Leukocytes were minimal or absent in this phase.

The estrus phase was characterized by an abundance of large, non-nucleated cornified epithelial cells arranged in clumps and sheets, showing angular and irregular shapes with faint “ghost” nuclei (Figures 1C, 1D, 2A, 2B). This phase corresponds to the period of ovulation and sexual receptivity.

The short-lived metestrus phase exhibited a marked increase in leukocytes, predominantly neutrophils, which appeared as small, round cells with multilobed nuclei (Figures 3A, 3B, 4A, 4B). Cornified epithelial cells were fewer and scattered. Occasional ruptured neutrophils were noted, emphasizing the fragile nature of leukocytes during smear preparation.

In the diestrus phase, leukocytes were the predominant cell type, diffusely scattered across the smear (Figure 4C). Epithelial cells were fewer, mainly nucleated, indicating a quiescent phase of the reproductive cycle.

Table 1 summarizes the cellular characteristics and typical duration of each estrous phase based on unstained vaginal cytology. The average estrous cycle duration was approximately 4 to 5 days, consistent with established literature.

**Table 1:** Summary of estrous cycle phases based on unstained vaginal cytology

Estrous phase	Duration (hours)	Predominant cells in unstained vaginal smears	Fertility status
Proestrus	10–14	Small, rounded nucleated epithelial cells, minimal leukocytes	Pre-ovulatory phase
Estrus	24–48	Large, non-nucleated cornified epithelial cells in sheets/clumps	Ovulatory, receptive phase
Metestrus	6–8	Increased leukocytes (neutrophils), fewer cornified epithelial cells	Post-ovulatory phase
Diestrus	48–72	Predominantly leukocytes (neutrophils, lymphocytes), few epithelial cells	Quiescent, non-receptive

Unstained vaginal cytology smears provided sufficient detail to reliably distinguish all phases of the estrous cycle without requiring staining, supporting the utility of this rapid and cost-effective method for reproductive monitoring in laboratory settings.

## Discussion

Summary of findings

This study confirms that unstained vaginal cytology is an effective and reliable method for staging the estrous cycle in female albino Wistar rats. The cytological changes observed during the proestrus, estrus, metestrus, and diestrus phases closely match the established patterns observed in stained smears, validating unstained cytology as a practical and cost-effective alternative for estrous cycle assessment in rodent models [6,7,8].

Comparison with previous studies

Our findings are consistent with previous studies that identified key cytological markers across the estrous cycle. The predominance of nucleated epithelial cells in proestrus, cornified epithelial cells in estrus, and leukocyte infiltration in metestrus and diestrus phases are in alignment with those observed in traditional stained smears [9,10]. These observations support the use of unstained vaginal smears as a reliable diagnostic tool, corroborating the findings of Goldman et al. [1] and Ajayi and Akhigbe [11,12], who reported similar patterns using stained smears for estrous cycle staging.

Advantages of unstained cytology

Unstained vaginal cytology provides a rapid, economical method for estrous cycle staging, requiring minimal reagents and technical skill. This technique is particularly beneficial for laboratories with limited resources, as it avoids the time and cost associated with staining procedures. Furthermore, unstained cytology allows for immediate slide examination, preventing degradation and minimizing artifacts commonly seen in stained smears. These advantages make it a valuable tool for reproductive and toxicological studies in resource-constrained environments [9,12].

Limitations and challenges

Despite its clear advantages, unstained cytology has certain limitations. The lack of color contrast may obscure fine cellular details, potentially leading to observer variability and misinterpretation, especially by less experienced personnel. The absence of staining may also make it difficult to distinguish subtle cellular features, which are crucial for accurate phase identification [13,14,15]. Therefore, training of personnel and potential use of digital image analysis tools could enhance the method's reliability and reduce observer bias.

Future directions

To improve the reliability of unstained cytology, future research should focus on inter-observer reproducibility and explore the potential of automated image analysis techniques to enhance the accuracy and objectivity of cytological assessments. Further studies could also correlate cytological staging with hormonal assays to validate this method's accuracy and provide deeper insights into estrous cycle physiology. Expanding the application of this technique to other rodent species or strains would help broaden its utility in both reproductive research and toxicological evaluations [16,17].

This study establishes unstained vaginal cytology as a viable, low-cost method for estrous cycle staging in albino Wistar rats. The method's simplicity and cost-efficiency could make it a valuable tool in both laboratory and field settings, particularly where resources are limited. Future work should focus on improving the precision of cytological interpretation through technological advancements and cross-validation with hormonal data.

Unstained vaginal cytology is a rapid, reliable, and cost-effective method for staging the estrous cycle in female albino Wistar rats. The technique accurately identifies the phases of the cycle without the need for staining, making it particularly valuable in laboratories with limited resources. Its adoption could significantly enhance reproductive and toxicological research by providing an accessible, effective means of monitoring estrous cycles in rodent models.

## Conclusions

Unstained vaginal cytology proves to be an efficient, cost-effective, and reliable method for accurately staging the estrous cycle in female albino Wistar rats. By successfully identifying the distinct phases of the cycle - proestrus, estrus, metestrus, and diestrus - this technique offers a practical alternative to traditional stained smears, making it particularly valuable for laboratories with limited resources. The adoption of unstained cytology could enhance the accessibility of reproductive and toxicological research, particularly in settings where rapid, low-cost techniques are crucial. Further validation studies, including inter-observer reproducibility and integration with hormonal assays, are recommended to strengthen its reliability and expand its application across various rodent models and experimental conditions.
